# Epizootiological monitoring of wolf helminths in Northern and Central Kazakhstan

**DOI:** 10.14202/vetworld.2024.1648-1654

**Published:** 2024-07-30

**Authors:** Rabiga Uakhit, Ainura Smagulova, Lyudmila Lider, Sergey Leontyev, Vladimir Kiyan

**Affiliations:** 1 Laboratory of Parasitology, Department of Veterinary Medicine, S. Seifullin Kazakh Agrotechnical Research University, Astana, Kazakhstan; 2 Laboratory of Biodiversity and Genetic Resources, National Center for Biotechnology, Astana, 010000, Kazakhstan

**Keywords:** *Echinococcus*, epidemiology, helminth prevalence, Kazakhstan

## Abstract

**Background and Aim:**

Wolves (*Canis lupus*) play a role in nature, including the regulation of the number of ungulates and the use of dead animals. In addition, wolves are a natural link and carrier for the spread of many parasitic invasions. Hence, the main task in preventing the spread of parasitic invasions is to regulate the wolf population. This study aimed to monitor the endoparasitological fauna of wild wolves inhabiting Northern and Central Kazakhstan.

**Materials and Methods:**

Overall, 81 wolves were investigated for parasitic worms using the K. I. Scriabin method. Wolf intestinal materials were collected from the following six regions: North Kazakhstan, Pavlodar, Kostanay, Akmola, Ulytau, and Karaganda. The genetic diversity of the parasites was identified using a polymerase chain reaction with specific primers. After data collection, a comprehensive statistical analysis was performed.

**Results:**

Several helminth types were identified in wolves, including *Echinococcus granulosus*, *Taenia hydatigena*, *Mesocestoides* spp., *Toxascaris leonina*, *Trichinella nativa*, *Alaria alata*, and *Dirofilaria repens*. Based on the results of this study, young male wolves aged 1–4 years were the most vulnerable to helminthiasis. Wolves living in steppe and semi-desert regions are often exposed to helminth infections. The prevalence of *T. nativa* in the wolves was 20.4%. This study also revealed the presence of echinococcosis among wolf populations in Karaganda and Kostanay, with prevalence rates of 4.1% and 4.7%, respectively. The overall prevalence of tapeworms in wolves was 54.3%.

**Conclusion:**

This study highlights the significance of understanding the potential risks associated with helminth infections in wild carnivores because helminths can act as disease reservoirs and pose a threat to humans, livestock, and other wild carnivores. These results can contribute to the development of effective control and management strategies for helminth infections in wolves, which can infect humans and livestock.

## Introduction

The gray wolf (*Canis lupus*) is ubiquitous in Northern and Central Kazakhstan [1]. Wolves, which are large mammals and forest cleaners, have varied diets. Therefore, wolves are hosts of parasites that are transmitted among canids, including prey [2, 3]. Thus, wolf-parasite associations can influence the population dynamics and ecological functions of wolves and their prey [4]. Geographic host range, population density, and body size are general indicators of parasitic richness across a wide range of taxa [5]. Thus, the parasitic communities of gray wolves provide a valuable system for understanding the role of parasites in host regulation and predator-prey dynamics.

A literature review of the studies compiling the final list of wolf-parasitic helminths revealed a comprehensive picture. A total of 72 helminth species belonging to 40 genera have been reported to infect wolves, with 93% detected in the gastrointestinal tract during necropsy [6]. Among them, 28 species of nematodes, 27 species of cestodes, 16 species of trematodes, and one acanthocephalan have been identified [7–10]. The most prevalent helminth is the tapeworm, *Taenia hydatigena*, which occurs at a relative frequency of 30% in all zoogeographic regions. The related tapeworm *Echinococcus granulosis* also showed a high prevalence (>19%). In tundra wolf populations, the dominant helminth species (73.9%) is the roundworm *Toxascaris leonina* [11–14].

It is extremely important to understand that wolves play a special role in the conservation and spread of natural invasions [15]. Wolf worms undergo several stages of their lifecycle, including the egg, larval, and adult stages. Most worms leave their host bodies during reproduction and move to the external environment. Consequently, the soil becomes contaminated with helminth eggs, which is one of the factors contributing to the spread of the invasion. The soil has properties favorable for maintaining the viability and preserving the invasive character of these worms. This is a significant concern because it determines the possibility of the transmission of the infection to humans.

Therefore, epizootic monitoring is becoming increasingly important [16]. Epizootic surveillance was conducted on a regional scale in Northern and Central Kazakhstan, where wolf carcasses were obtained [17]. According to Kazakhstani legislation, wolves are considered a species of animal whose populations are subject to regulations [18]. Control measures have been introduced to protect public health from diseases that can affect farms and other domestic animals [19]. In addition, regulating the number of such animals helps prevent damage to the environment and avoids significant damage to agricultural activities.

Studies conducted in Kazakhstan on the invasion of wild carnivores confirm the data described above [20–23]. The previous studies have established infestations of wolves with roundworms *Trichinella nativa* and *Dirofilaria repens* [24, 25], trematode *Alaria alata* [26], and cestodes *T. hydatigena* and *Echinococcus granulosus* [27].

The goals of this study were as follows: (1) To determine the type of helminths infecting wolves in Northern and Central Kazakhstan and (2) to assess the level of invasion and prevalence of helminths. We achieved these goals using 5 years of material collection (2019–2024), a combination of field and laboratory methods, and statistical analyses. Finally, we evaluate our approach for the benefit of future studies and its application in other fields.

## Materials and Methods

### Ethical approval

The study was approved by the Animal Ethics Committee (protocol No.1, dated July 24, 2019). This study adhered to the World Medical Association Code of Ethics (Declaration of Helsinki) for Animal Experimentation (http://ec.europa.eu/environment/chemicals/lab_animals/legislation_en.htm), which ensured that all animal procedures were performed ethically.

### Study period and location

The study was conducted from January 2019 to April 2024. The samples were collected from different regions of the Republic of Kazakhstan. The projects involved the use of biomaterials obtained from wolves and were conducted in the Parasitological Laboratory of the Faculty of Veterinary Medicine at S. Seifullin Kazakh Scientific Research Agrotechnical University.

### Parasitological studies

**Figure-1:**
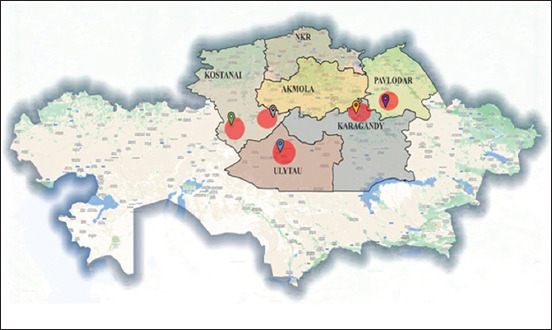
Map of the distribution of detected helminths in Northern and Central Kazakhstan [Source: The map was generated using the QGIS Version 3.18.0 program].

This study analyzed the digestive tracts of 81 wolves for the presence of parasites. Materials were collected from six regions: North Kazakhstan (97,993 km²), Pavlodar (124,755 km²), Kostanay (196,001 km²), Akmola (146,219 km²), Ulytau (188,936.61 km²), and Karaganda (239 045 km²). The geographic locations where the wolves were captured are shown in detail on the map in Figure-1. Full examination of the internal organs of wolves for the presence of parasitic worms was conducted using the K. I. Scryabin method [28]. To identify the taxonomy of these parasites based on their morphological characteristics, available guides and atlases were consulted [29, 30].

### Molecular genetic studies

Molecular genetic studies were conducted at the Laboratory of Biodiversity and Genetic Resources of the National Center for Biotechnology to confirm the species’ identity. The genetic diversity of the parasites was determined using a polymerase chain reaction (PCR) with specific primers for each helminth species [31–34]. Sequencing was performed to validate PCR results. This process ensured the accuracy and reliability of the study.

After data collection, a comprehensive statistical analysis was conducted. A diversity index was utilized to better understand the abundance and composition of helminths, which factored in the number of species present and the extent of their dominance.

## Results

This study considered several factors, including the age, sex, time of capture, and natural habitat of the wolves. Detailed information on the characteristics of the studied wolf samples is presented in Table-1.

**Table-1:** Characteristics of 81 studied wolves in northern and central Kazakhstan.

Parameters	Description	Number of cases out of the total	Infected cases	Prevalence, %	CI, 95%
Age groups	3-month-1 year	12	3	25	0.25 ± 0.487 (−0.237–0.737)
	1–4 years	25	22	88	0.88 ± 0.134 (0.746–1.01)
	5–8 years	4	4	100	1 ± 0 (1–1)
	Indeterminately	40	-	-	-
Gender	Male	30	21	70	0.7 ± 0.192 (0.508–0.892)
	Female	29	11	37.9	0.37 ± 0.284 (0.086–0.654)
	Indeterminately	22	-	-	-
Natural habitat	Forest-steppe	21	13	71.4	0.61 ± 0.261 (0.349–0.871)
	Steppe	27	18	74	0.66 ± 0.217 (0.443–0.877)
	Semi-desert	33	19	63.6	0.57 ± 0.22 (0.35–0.79)
Season	Autumn	3	2	66.6	0.66 ± 0.679 (−0.019–1.34)
	Winter	68	40	58.8	0.58 ± 0.146 (0.434–0.726)
	Spring	11	8	72.7	0.72 ± 0.305 (0.415–1.02)

CI=Confidence interval

**Figure-2:**
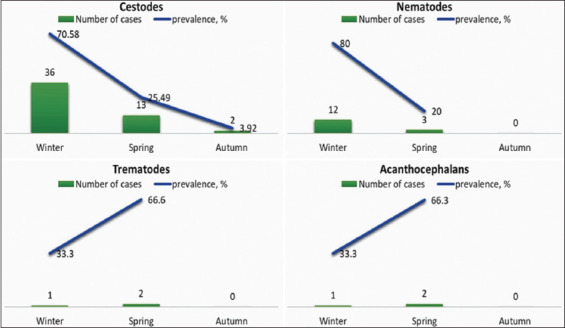
Distribution of the average number of parasites in the studied wolves.

The distribution of the average number of parasites from various families in the infected samples was calculated in relation to the capture time of year (Figure-2). This analysis provides valuable insights into the seasonal trends in parasite prevalence.

Figure-2 shows the identified helminths from four distinct families: Cestodes, nematodes, trematodes, and acanthocephalans. Among these, cestodes were the most prevalent, followed by nematodes, trematodes, and acanthocephalans. The total number of helminths detected was the highest for cestodes, indicating their dominant presence in wolves.

During winter, the samples exhibited a higher prevalence of cestodes, accounting for 70.5% of the total number studied. In contrast, in spring, the percentage of cestodes was significantly lower (4%). Similarly, the prevalence of nematodes was also higher during the winter (80%). In contrast, no helminths of the nematode family were found in spring samples. Trematodes and acanthocephalans, on the other hand, were found in equal numbers, predominating in the spring samples. Data on the number of helminths detected and the level of infestation are presented in Table-2.

**Table-2:** Helminthes found in wolves.

Place of capture	Number of studied		Species of helminths	Prevalence, %	Invasion, per sample

	Investigated	Infected			
Pavlodar region (Bayanaul district)	3	1	*Taenia hydatigena*	33.3	6
Karagandy region (Bukhar-Zhyrau district Karkaraly district)	48	30	*Taenia* species	62.5	7.5
		5	*Trichinella nativa*	10.41	30
		3	*Toxascaris leonina*	6.2	5.3
		2	*Echinococcus granulosus*	4.1	22.5
		2	*Acanthocephalans*	4.1	7.5
		1	*Alaria alata*	2	1
Ulytau region (Zheskagan district)	9	5	*Taenia hydatigena*	55.5	7.6
		2	*Trichinella nativa*	22.2	32.5
		2	*Toxascaris leonina*	22.2	3.5
Kostanai region (Zhangeldy district, Turgai district)	21	8	*Taenia hydatigena*	38.09	10
		6	*Trichinella nativa*	28.57	25.5
		1	*Echinococcus granulosus*	4.7	20
		1	*Dirofilaria repens*	4.7	1
		4	*Toxascaris leonina*	19.04	3.75
		1	*Acanthocephalans*	4.7	4

Three samples from the Pavlodar region were analyzed, one of which was infected with the cestode *T. hydatigena*. Forty-eight biomaterial samples from the Karaganda region were studied. Thirty of these samples were found to be infected with cestodes, five samples contained *T. nativa* nematodes, five samples contained *T. leonina*, two samples were infected with echinococcosis and acanthocephalans, and one sample tested positive for trematodes (*A. alata*). Nine samples from the Ulytau region were examined, five of which were positive for cestodes. The nematodes *T. nativa* and *T. leonina* were also found. Twenty-one biomaterial samples from the Kostanay region were examined. The following helminths were detected: *T. hydatigena* (8 positive), *T. nativa* (6 positive), *T. leonina* (4 positive), and one each of *E. granulosus, D. repens*, and *Acanthocephalans*.

The Shannon index was used to summarize the information on the abundance and species composition of helminths, considering the number of species and the degree of their dominance (Table-3). Based on the analysis of the diversity index, which was close to zero, we can infer that helminth species belonging to the cestode family are the most predominant among wolves in Northern and Central Kazakhstan. This indicates that these particular types of parasitic worms are more prevalent in this area than other helminth species.

**Table-3:** Shannon index on the number and species composition of helminths.

Species	Frequency	*p*_i_	*ln (p*_i_)	*p*_i_ * *ln (p*_i_)		
Cestodes						
*Taenia* spp.	48	0.64		−0.44		−0.28
*Echinococcus* spp.						
*Mesocestoides* spp.						
Nematodes						
*Trichinella nativa*	23	0.30			−1.20	−0.36
*Trichinella britovi*						
*Toxascaris leonina*						
*Dirofilaria repens*						
Trematodes						
*Alaria alata*	1	0.01			−4.60	−0.046
Acanthocephalans	3	0.04			−3.21	−0.12
Shannon diversity index (H):					0.834423	
Shannon equitability index (EH):					0.601909	

## Discussion

The investigation of the spread of parasitic worms in wolf populations identified several types of helminths, including the families *E. granulosus, T. hydatigena, Dipylidium* spp., and *Mesocestoides* spp.; roundworms *T. leonina*, *T. nativa*, and *D. repens*; and trematode *A. alata* [24–27]. The extent of wolf infestation by helminths was relatively high in the western part of the region (96.5%), whereas it was significantly lower toward the north-central part (65.2%). The average number of helminths was high and some infected animals carried multiple parasites. The intensity of helminth infestation in wolves was 7.6 copies per infected host [35].

Based on the information presented in Table-1, it appears that wolves between the ages of 1 and 4 years are the most vulnerable to helminth invasion. Furthermore, the data indicated that male wolves were more likely to be infected than their female counterparts, which could be attributed to their dominant social status within the pack. In addition, this study revealed that wolves living in the steppe and semi-desert regions were more likely to be affected by helminth infections. Among these regions, the semi-desert Karaganda area had the highest prevalence of helminth infections, suggesting that the environment in this region is particularly conducive to the spread and circulation of helminths. Overall, these findings suggested that age, sex, and habitat are important factors to consider when studying helminth prevalence in wolf populations.

### Trichinellosis

The prevalence of trichinellosis among wild predators in Northern and Central Kazakhstan is increasing. Of the 81 wolves examined, 17 (20.4%) were found to be infected with *T. nativa*. In this study, *T. nativa* was detected in three regions: Karaganda, with five positive samples; Ulytau, with two positive samples; and Kostanay, with six positive samples [24].

### Echinococcosis

Based on the findings of monitoring the prevalence of echinococcosis among wolf populations, positive samples indicated the presence of the disease in Karaganda (4.1%) and Kostanay (4.7%). Furthermore, the helminth infection rate per sample was 20 in the Kostanay region and 22.5 in Karaganda region. Sequence analysis of the cox1 and nad1 genes revealed that the type of echinococcosis present in the wolves was *E. granulosus*. Sequencing a portion of the mitochondrial genome enabled the determination of three haplotypes (Hp1, Hp2, and Hp3) of the pathogen in the studied sample. In addition, this study revealed that the dominant circulating *E. granulosus* genotype among wolves was G1, which is highly pathogenic to humans, livestock, and wild carnivores [27]. These findings suggest that wild carnivores, such as wolves, play a significant role as disease reservoirs [35].

### Alariosis

A previous study by Smagulova *et al*. [26] demonstrated the spread of trematodes in wolves and revealed that wolves are susceptible to infection by helminths belonging to the *A. alata* trematode family. This study not only highlighted the vulnerability of wolves to this specific type of parasite but also underscored the potential risks associated with its spread. These parasites are found in various hosts, including red foxes, wolves, raccoon dogs, and animals of the Felidae family, and are transmitted through intermediate hosts such as snails and frogs. The detection of *A. alata* in meat is not mandated, posing a significant risk of food-associated parasitic infections, particularly with the increasing popularity of game and organic meats processed without proper heat treatment [36, 37].

### Dirofilariasis

This is a rare case of helminth discovered in the heart cavity of a wolf and raises questions about its distribution in Kazakhstan. PCR was performed to identify the heartworm species found in the heart of a wild wolf using the species-specific primer SSU rRNA. A previous study by Uakhit *et al*. [25] showed that the nucleotide sequence of the studied species is *D. repens*. *D. repens* occurs only in the Commonwealth of Independent States and is common among the residents of Uzbekistan, Georgia, Armenia, Ukraine, Belarus, Russia, and Kazakhstan.

### Ascariasis

The study investigated 81 wolf samples and discovered that 6 of them (13.8%) tested positive for toxocariasis. The presence of roundworms has been confirmed in the Karaganda, Kostanay, and Aktobe regions. Through molecular genetic analysis, the roundworm was identified as *T. leonina*. Studies conducted worldwide have indicated the global distribution of this parasite, particularly in wild animals, which are considered reservoirs for *T. leonina* [36, 38, 39].

### Taeniasis

Our study revealed the significant prevalence of tapeworm helminths among wolves in Northern and Central Kazakhstan. This discovery is crucial, as it not only enhances our understanding of the health status of these canids but also provides a baseline for future ecological studies. This study showed that these parasites were found in four different areas and were commonly found in mixed infestations with other helminth types. The overall prevalence of tapeworms in wolves was 54.3%, indicating that many wolves in these regions were infected with these parasites. Interestingly, this study also found that wolves in the Karaganda and Ulytau regions were largely infested with taeniids, with prevalence rates of invasion of 81.7% and 90.9%, respectively.

Furthermore, this study underlines the vulnerability of wolves to helminth infections during winter, with infection rates 80% higher than those in other seasons. In other studies on wolf taeniasis, researchers have established a mixed infestation of cestodes by tapeworms in one individual. This study also detected five cestode species in wild wolves [12, 40].

This study revealed that wolves from different regions have varying degrees of susceptibility to helminth infections. For instance, in the Ulytau region, 27.27% of wolves were infected, with invasion intensities ranging from 1 to 45. In the Karaganda region, 20.4% of the wolves were infected, with an invasion intensity ranging from 2 to 63. In contrast, in the Kostanay region, the infection rate was 18.5%, with an invasion intensity ranging from 1 to 23. The percentage of infected wolves in the Pavlodar region was 33.3%, with an invasion intensity of 6.

Research on the prevalence of parasitic worms in wolf populations in various regions of Kazakhstan provides valuable insights into the factors that influence helminth infestation. This study underscores the importance of age, sex, and habitat when assessing the vulnerability of wolves to helminth invasions. The data indicate that wolves between the ages of 1 and 4 years are the most susceptible to helminth invasion, and male wolves exhibit a higher likelihood of infection, possibly due to their dominant social status within the pack.

Identifying specific helminth species such as *E. granulosus* and *T. hydatigena* provides essential knowledge for understanding the health risks of these parasites in wildlife and their potential transmission to humans and domestic animals.

## Conclusion

Overall, this study emphasizes the significance of monitoring and understanding the dynamics of helminth infestation in wolf populations for wildlife conservation and public health concerns related to zoonotic parasite transmission. These findings can inform targeted interventions to mitigate the risks of helminth infections in wildlife and interconnected ecosystems.

A more in-depth genetic analysis of the identified helminth species could provide additional information regarding their transmission patterns and potential impact on human and animal health. Long-term monitoring of wolf populations and their helminth infections could provide insights into the dynamics of these parasites over time, including potential fluctuations in prevalence, emergence of new strains, and effectiveness of control measures. Addressing these areas would contribute to a more holistic understanding of helminth infections in wolf populations and have implications for wildlife conservation, veterinary medicine, and public health initiatives.

The findings of the present study provide valuable insights into the ecology and health of wolves in this region. These results can contribute to the development of effective control and management strategies for helminth infections in wolves, which can infect humans and livestock.

## Authors’ Contributions

VK: Conception and design of the study, drafted, revised, and finalized the manuscript for submission, analyzed the data, and performed statistical analyses. RU and AS: Performed DNA extraction and PCR, interpreted the data, and drafted the manuscript for submission. LL and SL: Collected samples and performed parasitological isolation and typing. All authors have read, reviewed, and approved the final manuscript.
